# A Jeopardy-Style Review Game Using Team Clickers

**DOI:** 10.15766/mep_2374-8265.10485

**Published:** 2016-10-25

**Authors:** John Cusick

**Affiliations:** 1 Associate Professor, Department of Pharmaceutical and Biomedical Sciences, California Northstate University College of Pharmacy

**Keywords:** Active Learning, Problem-Based Learning, Jeopardy Review Game, Team Clickers, Student Engagement

## Abstract

**Introduction:**

Gaming has been used for many years as a useful supplemental instruction method in the biomedical sciences. However, the effective use of games can be challenging in a large classroom setting due to the difficulty of engaging all students. The goal of this exercise was to design an interactive way to review material that promoted small-group discussion in a large class of 104 students. A review game comparable to Jeopardy was utilized that enabled small teams to engage in friendly competition, using team clickers and a team leaderboard that tracked the team scores in a lowstakes, fun environment.

**Methods:**

Individual team clickers were used with a Jeopardy-style review game. TurningPoint served as the audience response system. Nineteen preassigned teams comprised of five to six students per team considered all questions during the closed-book review game. Discussion within teams was encouraged prior to each team's selection of an answer using the team clicker. A team leaderboard tracking the team scores was periodically displayed throughout the exercise, which helped keep the students engaged in a fun and relaxed atmosphere, as no extra points were awarded to the winning team.

**Results:**

The review game was enthusiastically received by the students and was frequently cited as an aspect the students enjoyed most about the course. The review game was also positively received by both pharmacy faculty and medical school faculty of the university when presented as a teaching tool during a faculty development session.

**Discussion:**

Although this resource was intended to serve as a serious game to review immunology topics for third-year pharmacy students, it can be used to teach a variety of science topics within the medical education setting. Similarly, while this review game was used within the context of team-based learning pedagogy, it can also be used in a traditional classroom setting, with preassigned teams for the review game.

## Educational Objectives

By the end of the review game, the learner will be able to:

1. Discuss review material associated with the biomedical sciences in a fun and interactive format.
2. Solve practice exam questions within small teams of five to six students, despite a class size of over 100.
3. Use effective communication skills within a group with regard to both sharing and justifying one's opinion of the correct answer and listening and considering the opinions of others.

## Introduction

Gaming has been used for many years as a useful supplemental instruction method in the biomedical sciences.^1,2^ However, the effective use of games can be challenging in a large classroom setting due to the difficulty of engaging all students. Teams containing a large number of students tend to be dominated by more confident or outgoing individuals. Conversely, introverted or less confident students are more hesitant to speak in large teams and therefore do not receive the same beneficial active-learning educational experience as their more outgoing classmates. I had previously incorporated games as an engaging method to review material in class sizes of 30–50 students. Teams of approximately 15 students took turns answering practice exam questions, with scoring used to provide friendly but low-stakes competition. Each team selected a team leader who was responsible for facilitating discussion and soliciting input from all members of the team prior to providing a final answer for the team. With class sizes of 30–50, I could stand in front of a group of approximately 12–15 students and verify that all students were actively engaged. To encourage engagement, I occasionally reminded particular students to join the discussion or prompted team leaders to seek input from all team members prior to providing the team's final answer. Students enjoyed these games as an active method to review material while promoting self-assessment, active learning, and teamwork skills. Upon becoming a course director for a class of 104 pharmacy students, I realized a new approach would be needed to promote active engagement of all students in a review game.

The goal of this exercise was to design an interactive way to review material that promoted small-group discussion in a large class of 104 students. A review game comparable to Jeopardy was utilized that enabled small teams to engage in friendly competition, using team clickers and a team leaderboard that tracked the team scores in a low-stakes, fun environment. Variations of Jeopardy review games have been used successfully in a variety of settings to promote student engagement.^3–9^ The Jeopardy review game discussed in this report differs significantly from previous reports in that it utilizes team clickers and a team leaderboard to improve the engagement of all students in a very large classroom setting.

## Methods

The attached Jeopardy review game instructional guide (Appendix B) is intended to assist the reader in using and modifying the attached blank template (Appendix A) for personal use. The attached review game example template (Appendix C) serves as an example of how the Jeopardy review game can be constructed. Although the review game was used in a course that utilized team-based learning (TBL) as its teaching methodology, it should be emphasized that this Jeopardy-style review is applicable to the traditional classroom setting, provided that teams are preassigned in some manner. Students were preassigned into 19 teams at the beginning of the course; each team contained five or six students. TurningPoint was used as the audience response system, and a Microsoft Excel Participant list specific to the 19 team clickers (Appendix D) was loaded onto the classroom computer. Practice exam questions were loaded into the TurningPoint file, with differing point values assigned to the questions in relation to their relative difficulty ($100, $200, etc.). A game board was created using Microsoft PowerPoint as described the on iSpring website (http://www.ispringsolutions.com/blog/how-to-make-a-jeopardy-game-in-powerpoint/). Hyperlinks were used to link the game board to individual questions. Each question was followed by an answer slide that displayed the correct answer. For conceptually difficult questions, extra informative slides containing figures from the course textbook were incorporated after the answer slide to clarify points before moving on to the next question. A team leaderboard tracking the team scores was included as the last slide in the attached blank and example template files (Appendices A & C, respectively). All of the answer slides contained a hyperlink labeled *SCORE* that was linked to the team leaderboard. The team leaderboard itself contained a hyperlink labeled *Game Board* that led the audience back to the game board. Trial runs were performed in the classroom to verify that both the team clickers and the team leaderboard were functioning as expected. Each team clicker had the team number clearly displayed to facilitate quick distribution of the clickers to the teams at the start of the review game.

Several modifications were made to the Jeopardy game to make it amenable to incorporating input from many teams in a large classroom. First, all of the questions were systematically considered by all teams, as permitting students/contestants to control the game board, as occurs on the television show, would be problematic due to the large class size. This format also made the Daily Double and Final Jeopardy options impractical. Second, questions were formatted similarly to exam questions, and therefore, students selected the best answer from multiple-choice questions using their clickers, rather than providing answers in the form of a question, as occurs on the popular television show. Additionally, points were not deducted for incorrect answers, as this would make scoring problematic and unnecessarily increase anxiety among students.

A 30-second countdown was initiated once half of the teams had submitted their answers. The more challenging questions (worth $400 and $500) offered an opportunity to walk around the room to ensure engagement by all students, as the amount of time required for team discussions was substantially longer than for less challenging questions. Displaying the range of team answers via the audience polling histogram permitted time for a brief reflection by team members prior to advancement to the answer slide. Further explanation could be provided after the answer slide was displayed if numerous teams missed a question or if specific questions were raised by students. The team leaderboard was typically surveyed after the $400 and $500 questions as the team leader list often changed significantly after these heavily weighted questions. The team leaderboard was displayed for no more than 5 seconds to impede students in ascertaining the results of other teams, as this could lead to a less positive atmosphere. Similarly, the team leaderboard was deleted from the review game file prior to distributing the file to students for use as a study guide.

The attached blank template (Appendix A) can be implemented effectively in a variety of classroom settings, yet it works particularly well for TBL. The immunology course met for 2-hour sessions twice a week using TBL as the teaching methodology. Therefore, an individual Readiness Assurance Test (iRAT), a team Readiness Assurance Test (tRAT), and application exercises were used for each individual session, as previously described for TBL.^10^ The Jeopardy review application was used approximately every 2 weeks and was always used during the last session before an exam. The schedule for a typical 2-hour TBL session using the Jeopardy review application is as follows:

• 10:00–10:25: iRAT and tRAT covering new subject material assigned in the reading.
• 10:25–10:40: If necessary, a brief lecture or explanation could be used if many students missed a particular iRAT question or if students requested an explanation for a particular topic in the preassigned reading. Otherwise, this time could be devoted to the application exercises.
• 10:40–11:20: first application exercise covering new material from the preassigned reading.
• 11:20–12:00: the Jeopardy review game as a second application exercise, covering subject material for four TBL sessions, including the preassigned reading for the current TBL session.

## Results

The review game using team clickers was enthusiastically received by the students. Students demonstrated a high level of engagement and interest during the exercise, and numerous positive comments regarding the game were received in the course evaluations. Seventy-eight of the 104 students provided feedback when asked on the course evaluations, “What did you like most about the course?” In addition to several comments that generally voiced support for “fun exercises,” 13 of the 78 comments (16.7%) specifically referenced the Jeopardy game as an aspect that they liked most about the course. The following are examples of specific student comments received in support of the Jeopardy game:

• “Jeopardy games were really helpful and helped bring all the concepts together.”
• “Jeopardy was very fun, competitive, and helpful!”
• “I enjoyed the Jeopardy sessions the most; they provided us with the chance to test our knowledge before exams.”
• “His Jeopardy games were a wonderful way of reviewing for the exams.”
• “Loved the Jeopardy games—helped me evaluate where my weaker points were.”

In contrast, 58 of 104 students provided feedback when asked, “What can be done to improve the course?” Only one answer specifically referenced the Jeopardy game, and it did so in a positive light:

• “Maybe more Jeopardy games the class was one of my favorites.”

A presentation of the Jeopardy review game to both pharmacy and medical school faculty was well received, with faculty from both colleges requesting the templates for the review game after the presentation.

## Discussion

The use of team clickers in the context of a Jeopardy-style review game was very positively received by students and served as a useful application exercise to review material periodically. The use of team clickers with an audience response system that displays a team leaderboard serves as a valuable tool to promote active learning in a large classroom setting. Team clickers were previously incorporated with gaming for a modified version of Who Wants to Be a Millionaire used to review concepts for medical students,^11^ and the attached blank template (Appendix A) provides an additional resource to combine team clickers with gaming. The use of team clickers has the potential to be expanded to other applications besides review games, for example, as another mechanism to provide simultaneous reporting in TBL application exercises.

Although the review game provided serves as a useful tool to review material in any type of biomedical science course that uses the traditional lecture format, this review game works especially well for TBL. First, a TBL class will already have teams selected. Second, TBL tends to look forward, as the administration of iRATs, tRATs, and application exercises on a daily basis serves to keep students on track with new reading material. Yet it is at times beneficial to look backwards to review material or to tie concepts together. In addition to being scheduled immediately before an exam, review games were also scheduled on days when there was not a particularly challenging new concept introduced in the preassigned reading. The periodic use of the review game, once every fourth session on average, prevented the game from becoming too long and permitted time for an additional distinct type of application exercise specific to the preassigned reading.

Although the review game was used as a TBL application exercise, it can also be used in a traditional classroom setting, with preassigned teams for the game. New teams at our institution are assembled every semester with an effort to avoid creation of superteams that would result in a negative atmosphere through domination. If a traditional classroom incorporates the attached review game, it might be beneficial to randomize teams, and adjust them if necessary after indicators of student performance are available, in order to promote enjoyment through a more level playing field. Conversely, the use of student-selected teams could lead to domination by teams comprised of competitive individuals, which would likely lead to disengagement of other teams that did not feel that they had a realistic chance of succeeding.

One drawback of any review game is that at least one individual or team will finish last. The use of teams lessens the negative impact of finishing last in comparison to games in which individual students compete in front of an audience of their peers. The team leaderboard was displayed for only a few seconds to minimize the time that students had to study the results of teams other than their own. The lack of additional points awarded to the winning team was an additional effort to decrease anxiety associated with grades. Although the necessity of one team finishing last is an unfortunate consequence of any game, the positive results of engaging students in a fun and collaborative exercise appear to have outweighed this disadvantage, as numerous students voiced support for the Jeopardy review game in the course evaluations and no negative comments regarding the game were received.

One additional drawback of this review method is the need to create hyperlinks for each question, yet the amount of time required can be minimized if hyperlinks are introduced only after the slide order is finalized.

There are several ways the game can be improved for future use. Alternating the student responsible for using the clicker to submit answers for the team would provide additional learning experiences, as it was observed that individual team members repeatedly held the clicker and submitted answers for their respective teams throughout the duration of the course. For challenging questions that receive mixed opinions from team members, the opportunity to mediate discussion prior to submitting the final answer for the team offers an opportunity to gain leadership skills. Since the teams are very small and each student within a team is associated with a number between 1 and 6, the use of an animated rolling die on the projected screen at the start of the exercise could serve as a tool to designate team leaders responsible for submitting the team answer via the team clicker. Ensuring each student serves as the team leader at least once during the semester would enhance the educational experience received by all students, similar to what has been proposed in a previous report.^7^ An additional improvement to the game would be to designate officially in the syllabus when Jeopardy-style review games will occur and to include the topics to be covered during that particular review game. This could serve as a potential motivating factor, especially for teams that have been struggling in previous review games.

The Jeopardy review game described differs from previous reports in order to make the game more efficient and engaging for large classes containing many teams of students. For example, most previous implementations of Jeopardy review games permitted the audience to control the game board,^3,6–9^ as is done in the television show game format, based on which team was the first to have a student raise his or her hand^3^ or through the use of buzzers.^4,5,9^ Yet with a large class comprised of 19 teams, determining which student raised a hand first would unfeasible, and the use of buzzers would require implementation of additional technology in the classroom. In an effort to streamline the game, questions on the game board were considered sequentially by all teams, negating the need for determining how students would control the game board. Simplifying the traditional television show game format, which involves only three individual competitors, also significantly decreases the time required to explain and implement the rules. The removal of the Daily Double feature after it was determined to be unnecessarily distracting in a separate study^6^ is another example of how streamlining the Jeopardy format can prevent the game from slowing down needlessly, leading to student frustration or disengagement, especially in large classes containing many teams.

The use of team clickers that tracked student answers with a team leaderboard significantly distinguishes this Jeopardy review game from previously published educational Jeopardy games, as all 19 teams of students were able to submit an answer for each question after engaging in small-group discussion. In contrast, individual teams would be able to provide an answer for only one out of every 19 questions on average if formats from previous games were used in which questions were answered by individual teams, based on which student raised a hand first^3^ or through the use of buzzers.^4,5,9^ Permitting each team to submit an answer for each question encourages active engagement of all teams throughout the entire game, as all questions throughout the game culminate in the final team standings at the conclusion of the game.

An additional advantage in using the team clickers during the Jeopardy review game is the ability to keep team sizes small despite a class size of over 100 students. The majority of previously published reports of Jeopardy review games were conducted in classes with fewer than 60 students^4–9^ and even as few as eight^4^ or nine students.^6,9^ One Jeopardy review game was conducted in a class that averaged 160 students subdivided into 16 teams with approximately 10 students per team.^3^ Individual teams answered questions based on which student raised a hand first. Although that game was very well received by students, the Jeopardy game described in this report would offer significant advantages. First, the use of team clickers and a team leaderboard would allow each team to submit an answer for each question, as described previously. Secondly, the use of team clickers would offer the opportunity to break the approximately 160 students into smaller teams of five to six per team, rather than 10 students per team, as the ability to determine which student raised a hand first would likely be a limiting factor in the ability to subdivide the 160 students into more than 16 teams. A previous report noted that increasing the size of a team from eight students to about 10 had a noticeable effect, as some students were less likely to speak on the slightly larger team.^7^ The ability to limit teams to five to six students each, despite more than 100 students being in the class, through the review game described here enhances the educational value individual students receive when being engaged in small-group discussion during review games. Finally, the use of team clickers also offers the ability to promote leadership experiences, as the student responsible for mediating small-group discussions prior to using the team clicker to submit the answer for the team can be rotated throughout the semester.

## Appendices

A. Review Game Blank Template.ppt
B. Review Game Instructional Guide.ppt
C. Review Game Example Template.ppt
D. Review Game Participant List.xlsx All appendices are peer reviewed as integral parts of the Original Publication.
